# Neuroenhancement with Vitamin B12—Underestimated Neurological Significance

**DOI:** 10.3390/nu5125031

**Published:** 2013-12-12

**Authors:** Uwe Gröber, Klaus Kisters, Joachim Schmidt

**Affiliations:** 1 Academy of Micronutrient Medicine, Zweigertstr. 55, Essen 45130, Germany; E-Mails: kisters@annahospital.de (K.K.); prof.schmidt.dd@t-online.de (J.S.); 2 St. Anna Hospital, Hospitalstr. 19, Herne 44649, Germany

**Keywords:** Vitamin B12, elderly, Vitamin B12 deficiency, diagnostic of Vitamin B12 deficiency, brain atrophy, neuroenhancement

## Abstract

Vitamin B12 is a cofactor of methionine synthase in the synthesis of methionine, the precursor of the universal methyl donor S-Adenosylmethionine (SAMe), which is involved in different epigenomic regulatory mechanisms and especially in brain development. A Vitamin B12 deficiency expresses itself by a wide variety of neurological manifestations such as paraesthesias, skin numbness, coordination disorders and reduced nerve conduction velocity. In elderly people, a latent Vitamin B12 deficiency can be associated with a progressive brain atrophy. Moderately elevated concentrations of homocysteine (>10 µmol/L) have been associated with an increased risk of dementia, notably Alzheimer’s disease, in many cross-sectional and prospective studies. Raised plasma concentrations of homocysteine is also associated with both regional and whole brain atrophy, not only in Alzheimer’s disease but also in healthy elderly people. Clinician awareness should be raised to accurately diagnose and treat early Vitamin B12 deficiency to prevent irreversible structural brain damage.

## 1. Introduction

**Figure 1:**
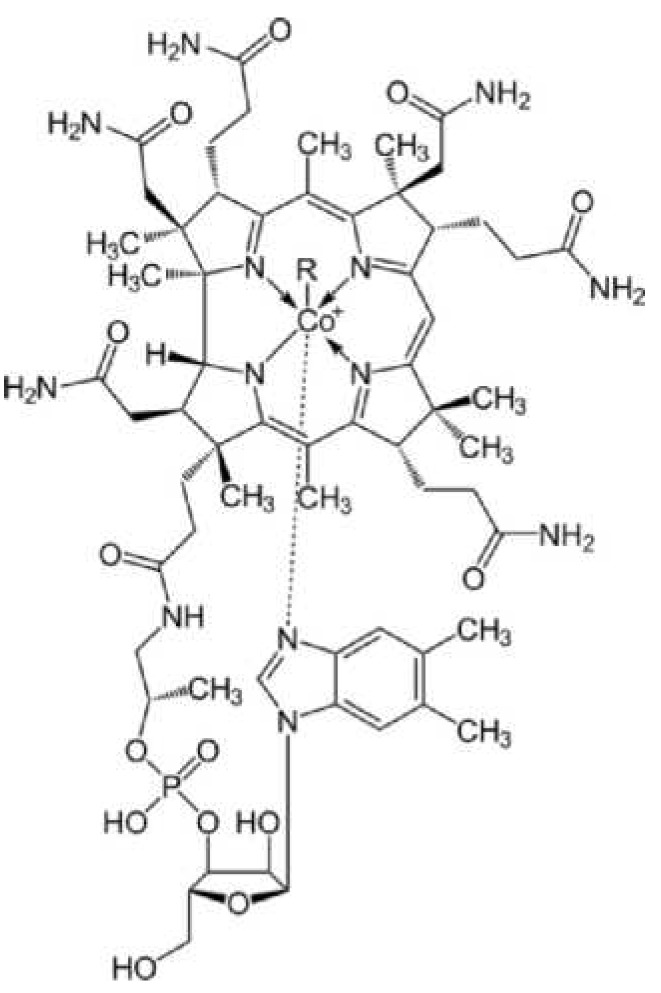
Chemical structure of cobalamin.

Vitamin B12 is an essential water-soluble vitamin that is vitally important in haematopoiesis, nervous system functions, maintenance of intact gastrointestinal mucosa and regulation of numerous other B12-dependent metabolic processes. The structural framework of Vitamin B12 is based on the corrin ring system comprising four reduced pyrrole rings and a central cobalt atom (Figure 1).

Substitution at the sixth ligand of the cobalt atom

- with CN results in cyanocobalamin,
- with OH- results in hydroxycobalamin,
- with H_2_O results in aquocobalamin,
- with NO results in nitrocobalamin,
- with CH_3_ results in methylcobalamin,
- with 5-Desoxyadenosyl results in adenosylcobalamin.

Vitamin B12 is a collective term for these variously substituted corrinoids. The principal biochemical participants are two coenzyme forms of Vitamin B12 that are produced and activated in two separate cellular compartments: methylcobalamin in the cytosol and adenosylcobalamin in the mitochondria.

Methylcobalamin functions as a cofactor to methionine synthase, which catalyses the remethylation of homocysteine to methionine, whereby the methyl group comes from the 5-methyl-THF. As the most important coenzyme in methylgroup transfer, it plays a significant role in transfer of C1 bodies, e.g., for choline synthesis and regeneration of methionine from homocysteine (methionine synthase) with participation of 5-methyltetrahydrofolic acid for the formation of the C1 body THF. Methylation of the cobalamin takes place in the cytosol.

Adencosylcobalamin is an important cofactor in rearrangement reactions, for example, of d-methylmalonyl-CoA-mutase in degradation of odd-numbered carbon skeleton of amino acids (threonine, valine, methionine) and fatty acids (propionyl-CoA). If this reaction cannot take place due to a lack of cobalamin, methylmalonyl-CoA is hydrolized to methylmalonic acid and eliminated in the urine. The formation of adenosylcobalamin takes place in the mitochondria. This requires that the central atom Co3+ is reduced to Co1+ in a reaction dependent on NADH/H+ and FADH2. The remaining adenosyl is supplied by ATP.

Cobalamin contributes significantly to haematopoiesis, myelin synthesis and synthesis of epithelial tissue. As a coenzyme, it is also a principal component of fatty acid, carbohydrate and nucleic acid metabolism [1].

## 2. Vitamin B12 Deficiency Symptoms

A Vitamin B12 deficiency manifests in humans mainly as

- haematopoietic disorders, affecting in particular the formation of erythrocytes,
- neurological/psychiatric disorders and
- epithelial changes in the mucosa of the digestive tract [2].

The haematopoietic disorders and neurological sequelae have a special clinical relevance since they may be linked to severe or even life-threatening diseases. Whereas the haematopoietic changes are highly characteristic and are therefore primary indicators in diagnosis of a Vitamin B12 deficiency, the neurological disorders show a much greater range of variation and do not receive due recognition as effects of a B12 deficiency in clinical practice. Neurological disorders, however, are often the earliest and, in some cases, the only clinical symptoms of a functional Vitamin B12 deficiency. The incidence data vary. According to the IOM (Institute of Medicine, Washington, DC, USA), 75%–90% of persons with a clinically relevant B12 deficiency have neurological disorders, and in about 25% of cases these are the only clinical manifestations of the B12 deficiency [1,3,4]. The neurological disorders may occur together with the haematological changes or independently of them. On the whole, it can be assumed that about 60% of patients with pernicious anaemia will also manifest symptoms of a funicular myelosis. About one-quarter of the patients with confirmed Vitamin B12 deficiency and neurological disorders showed no haematological changes. Interestingly enough, there is also an inverse correlation between the severity of the haematological and neurological disorders [5]. The more severe the neurological disorders, the less significant the haematological changes, and vice versa. The causes of this are unknown.

### Vitamin B12 Deficiency: Wide Variety of Neurological Manifestations

The most prominent neurological symptoms are paraesthesias or skin numbness, hands or feet that have “gone to sleep”, unsteady gait and coordination disorders up to and including paralyses. These symptoms are expressions of funicular myelosis (funicular spinal cord disease, subacute combined degeneration (SCD) of the spinal cord) [6,7]. This condition develops as a result of combined degeneration of the lateral and posterior funiculi of the spinal cord due to defective myelin sheaths and is a demyelinating disease. Affected structures:

- posterior funicular tracts (which convey tactile perception and proprioception),
- cerebellar lateral funicular tracts (which also contribute to proprioceptive perception) and
- the medullary pyramidal tract (which contributes to motor control).

The resulting neuropathy is symmetrical and is more pronounced in the legs than in the arms. In most cases, this neuropathy takes the form of a sensorimotor peripheral polyneuropathy, although mononeuropathies (optical or olfactory), autonomous neuropathies (impotence, incontinence), and combined forms (myelopathy and neuropathy) are also possible [3,7]. Patients initially notice a paraesthesia, at first in the feet and sometimes the hands, which then spreads over the limbs in the course of the disease. The sensory disturbances are followed by motor disorders (muscle weakness, paralysis symptoms, and motor coordination disorders). The nerve conduction velocity is reduced in motor and sensory nerves. Left untreated, the worst-case outcome of this disease would be paraplegia. Also possible are damage to central nerve tracts and cerebral disorders resulting in psychiatric symptoms. Remarkably there have been some cases of funicular myelosis with normal Vitamin B12 serum levels but with metabolical Vitamin B12 deficiency [8].

The symptoms of the cerebral disorders vary and may include confusion, stupor, apathy, memory and judgment disorders or even psychoses, depressions and dementia. The cerebral disorders, albeit less frequent than the peripheral symptoms, are underestimated in practice. Catatonia is also described as a psychiatric form of Vitamin B12 deficiency. Age-related limitations of mental performance, progredient cerebral atrophy, and Vitamin B12 status show clinically relevant interrelationships. The clinical manifestations of B12 deficiency in infants and small children whose mothers suffered from Vitamin B12 deficiency require special attention. Such infants may develop severe haematological and neurological disorders with lasting harmful effects on child development [9].

The diseases and disorders mentioned above may be caused by a variety of factors (e.g., drugs), among which B12 deficiency is often recognized too late. For this reason, the possibility of a Vitamin B12 deficiency should always be considered when such neurological disorders occur. In these cases, treatment with Vitamin B12 leads to rapid symptom alleviation.

## 3. Diagnostics of B12 Deficiency

There is no “gold standard” for laboratory chemistry confirmation of a clinically relevant Vitamin B12 deficiency. In practice, diagnosis of a Vitamin B12 deficiency is primarily done by determining the serum Vitamin B12 level (serum cobalamin level). This is a low-cost test with limited specificity and sensitivity, particularly in persons with Vitamin B12 concentrations <400 ng/L. The normal serum levels based on modern laboratory chemistry methods are 200–1000 ng/L.

Levels <200 ng/L (<150 pmol/L) are sure signs of a B12 deficiency; but a functional B12 deficiency may also be present at levels under 450 ng/L. Persons with B12 concentrations within the reference range may already manifest clinical signs of a Vitamin B12 deficiency [10]. Characteristic examples are cases of clinically and MRI-confirmed severe funicular myelosis despite normal Vitamin B12 levels and prompt response to B12 substitution [11].

Further investigative work is therefore required in this borderline area. This includes:

### 3.1. Determination of Holotranscobalamin

In the blood, Vitamin B12 is bound mainly to the proteins transcobalamin and haptocorrin, whereby only the smaller portion bound to transcobalamin (about 10%–25%) is bioavailable and active [12]. This complex of transcobalamin and Vitamin B12 is called holotranscobalamin (holo-TC) and its level is reduced in Vitamin B12 deficiency. Levels <35 pmol/L are indicative of a deficiency of Vitamin B12, and levels from 35 to 50 pmol/L represent a “grey area”. A vitamin B12 deficiency is unlikely at levels >50 pmol/L. A reduced holo-TC level is considered the earliest marker for a B12 deficiency. Holo-TC is therefore considered a better marker than serum B12 for determination of B12 status. Theoretically HoloTC should be a better marker than serum B12 but the fact that its confounders are not well investigated, its short half-life in plasma (90 min) and the poor specificity of the HoloTC test should be acknowledged [13].

### 3.2. Determination of Methylmalonic Acid

Methylmalonic acid (MMA) is considered a highly sensitive functional indicator for a Vitamin B12 deficiency [14,15]. MMA is a metabolic product, the breakdown of which requires Vitamin B12. A lack of Vitamin B12 allows MMA levels to increase significantly. This also makes it possible to detect a functional B12 deficiency in which raised MMA levels and clinical symptoms of a B12 deficiency in the form of neurological and/or haematological disorders may occur despite normal serum cobalamin levels [14]. MMA can be measured in both serum and urine. Cases of impaired renal function, however, may also manifest raised MMA, for which reason the creatinine level should also be determined to confirm sufficient renal concentration capacity. The normal range for serum MMA is 50–300 nmol/L. MMA determination is a highly sensitive test that can confirm a diagnosis even in the early stages of a B12 deficiency and is also a suitable indicator for treatment response (B12 substitution). In contrast to determination of the serum cobalamin level, which is only sufficiently sensitive in cases of alimentary B12 deficiency and disturbed enteral absorption of Vitamin B12, determination of MMA also facilitates detection of less frequent dysfunctions in the Vitamin B12 transport path and intracellular synthesis of active adenosylcobalamin. A round-table discussion in 2010 on selection of the biomarkers for determination of B12 status in continued NHANES studies concluded that determination of serum B12 and methylmalonic acid (MMA) should be recommended for further studies [16].

Another indirect functional parameter of Vitamin B12 status is homocysteine. This potentially toxic amino acid results from demethylation of the essential amino acid methionine, which reaction requires Vitamin B12. A deficiency of Vitamin B12 leads to accumulation and thus raised blood levels of homocysteine. A serum homocysteine level >10 µmol/L thus indicates a possible deficiency of Vitamin B12. However, homocysteine is not a specific B12 deficiency marker, since deficiencies of Vitamin B6 and folic acid can also raise the homocysteine level.

Determination of methylmalonic acid and homocysteine are particularly recommended in cases of diagnostically unclarified B12 deficiency. A Vitamin B12 deficiency can be excluded with something approaching 100% certainty if levels of these metabolites are within the normal ranges [16].

## 4. Incidence of B12 Deficiency

The incidence of Vitamin B12 deficiency is underestimated in practice. This is due mainly to statements issued repeatedly regarding provision of a sufficient supply of Vitamin B12 to the populace. Representative data on current nutritional behavior in Germany is provided by the National Nutrition Survey II (2008) [17]. This survey also included vitamin consumption in Germany. The investigations revealed a deficient supply of Vitamin B12 in a considerable number of persons of different ages and sexes (Table 1).

**Table 1:** Number of persons with dietary Vitamin B12 intake below the reference ranges, expressed as percentages.

Sex	Age in years				
	19–24	25–34	35–50	51–64	65–80
Men	7.4	6.8	8.4	7.9	9.8
Women	32.7	26.4	24.5	23.0	26.3

Thus, the daily dietary intake levels alone reveal a risk of pathological B12 deficiency status in a considerable number of persons due to malnutrition.

However, not every case of insufficient vitamin intake leads to a vitamin deficiency. Insufficient vitamin intake is defined as intake of an amount below the reference daily vitamin intake amounts, but vitamin deficiency means development of clinically relevant, measurable disorders or characteristic deficiency symptoms due to insufficient intake. Nonetheless, any person with insufficient vitamin intake is also at risk for development of a functional vitamin deficiency. The incidence of Vitamin B12 deficiency confirmed by laboratory chemical analysis is reported as 5%–7% for younger persons [8]. Vitamin B12 deficiency is widespread in the elderly in particular and has been diagnosed in 10%–30% of “healthy” persons above the age of 65 [3,18]. Despite intake of Vitamin B12 in amounts in excess of the reference levels, a person may suffer from a functional B12 deficiency [10]. Vitamin B12 deficiency is considered to be a worldwide problem [3].

## 5. Causes of B12 Deficiency

The causes of Vitamin B12 deficiency are varied and can be classified in four groups:

1. Dietary deficiency
  - Due to insufficient dietary intake of Vitamin B12; risk groups include alcoholics, vegetarians/vegans and older persons.
2. Malabsorption (disturbed uptake of Vitamin B12)
  - due to lack of intrinsic factor or parietal cells, e.g., in cases of pernicious anaemia, atrophic gastritis, postgastrectomy syndrome
  - disturbed uptake of Vitamin B12 from food due to gastric acid deficiency (e.g., in cases of long-term intake of acid secretion inhibiting substances (proton pump inhibitors, H-2 blockers)
  - drug interactions (e.g., antibiotics, anticonvulsants, colchicine, metformin, N_2_O)
3. Intestinal diseases
  - intestinal resection, tropical sprue, Crohn’s disease, Zollinger-Ellinger syndrome, intestinal bypass, Imerslund-Grasbeck syndrome
  - bacterial overgrowth (e.g., helicobacter)
  - Fish tapeworm infestation
  - drug interactions (e.g., metformin, chronic exposure to N_2_O)
4. Disturbed utilization of Vitamin B12 and inborn errors of Vitamin B12 and metabolism [19] Malabsorption is the underlying cause in most cases.

### 5.1. Proton Pump Inhibitors (e.g., Omeprazole) and Vitamin B12

A number of prescription drugs can deplete vitamin B12 stores, particularly in older patients. Over 60% of sales of proton pump inhibitors are accounted for by gastrointestinal drugs. In patients whose stomach acid content has been reduced by long-term treatment with a proton pump inhibitor (e.g., omeprazole) due to peptic disease, special attention should be directed to Vitamin B12 intake. Proton pump inhibitors reduce gastric acid secretion and thus the intestinal release of Vitamin B12 from foods. Current research suggests that up to 40% of persons over 65 have an insufficient intake of Vitamin B12 (Vitamin B12 serum levels < 450 ng/L) [20]. Particularly problematical is the fact that a Vitamin B12 deficiency is often associated with a folic acid deficiency and significantly increases the risk for a hyperhomocysteinaemia (>10 µmol/L). Folate and Vitamin B12 play an important role in the function of the CNS. Both vitamins are involved in methionine-homocysteine metabolism. Methionine becomes activated to S-adenosylmethionine (SAM), which is indispensable for numerous methylation reactions. Disturbances of these processes are involved in neurodegenerative diseases.

**Figure 2:**
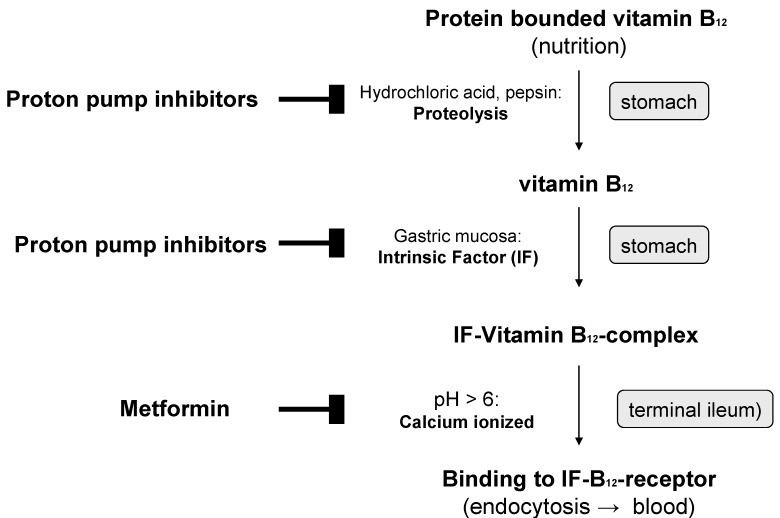
Inhibition of the active absorption of Vitamin B12 by proton pump inhibitors and metformin (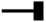 inhibition).

In older individuals, Vitamin B12 deficiency is due in most cases to insufficient production of gastric juice (achlorhydria). The main cause is inflammatory processes in the gastric mucosa, which develop primarily as a result of type B atrophic gastritis type B with subacidity and anacidity, as well as reduced intrinsic factor production. Protein-bound Vitamin B12 is therefore insufficiently released and absorbed (Figure 2). Cobalamins from food bound to protein are released in the stomach by hydrochloric acid and pepsin and bound to intrinsic factor (IF-B12 complex) in a pH-dependent reaction. Cellular cobalamin uptake in the mucosal epithelium takes place in the terminal ileum with the help of specific receptors of the brush-border membrane. The IF-B 12 complex binds to cubilin, which initiates calcium-dependent, receptor-mediated endocytosis together with another protein, megalin. Vitamin B12 uptake can also take place independently of the intrinsic factor by means of passive diffusion in the small intestine. This mechanism, however, is not very efficient, since only about 1% of the ingested Vitamin B12 dose is absorbed.

Reduced gastric acid production in the stomachs of older persons leads to alkalizing of the small intestine milieu, which eliminates the physiological barrier to microorganisms. Bacteria from lower intestinal segments can then increasingly enter the jejunum and ileum. Bacterial overgrowth with Clostridia and Campylobacter is accompanied by increased consumption of Vitamin B12 (conversion into inactive cobalamide) and by bacterial synthesis of substances that compete with the vitamin for receptors in the ileum mucosa. This further reduces the availability of Vitamin B12. In older persons (>65 years), atrophy of the gastric mucosa is frequently due to an infection with Helicobacter pylori. As many as 60% of elderly persons exhibit an increased infestation of the gastric mucosa with Helicobacter pylori and thus a high risk of developing chronic atrophic gastritis [2,21,22]. A regular supplementation of at least 100 µg Vitamin B12 daily in combination with folic acid and vitamin B6 is recommended for persons who regularly take proton pump inhibitors to reduce gastric acid secretion. Since the amount of actively absorbed Vitamin B12 depends on the availability of intrinsic factor (IF), some authors consider a maximum drug dosage (e.g., 1000 µg B12/day p.o.), thus allowing uptake of even small amounts of the vitamin through passive absorption [2].

### 5.2. Metformin and Vitamin B12

Metformin is used mainly in treatment of overweight type 2 diabetics since, in contrast to the insulinotrophic antidiabetic drugs, it does not generally initiate weight gain and/or hypoglycaemic attacks. In earlier clinical studies, Vitamin B12 malabsorption and a drop in Vitamin B12 serum levels by up to 30% were repeatedly observed under long-term treatment with metformin (Figure 2) [23]. Metformin leads to a disruption of calcium-dependent and receptor-mediated endocytosis of the intrinsic factor-Vitamin B12 complex in the terminal ileum [2].

These data are confirmed by the findings of current studies. In a case-control study, 155 diabetics who had developed a Vitamin B12 deficiency under treatment with metformin (average Vitamin B12 serum levels 148.6 ± 40.4 pg/mL (110 ± 30 pmol/L)) were compared with 310 control subjects who showed no deficiency of Vitamin B12 under the same medication. After adjustment for potential influencing factors, this resulted in a statistically significant correlation between the duration and dosage of metformin treatment and Vitamin B12 deficiency. Each dose increase by 1 g/day increased the risk of a Vitamin B12 deficiency by more than double (odds ratio 2.88; 95% confidence interval, 2.15–3.87; *p* < 0.001). In comparison with a treatment duration of less than 3 years, the odds ratio for a treatment duration of 3 years and longer was 2.39 (95% confidence interval, 1.46–3.91; *p* = 0.001) [23]. In another study of 165 type 2 diabetics, the influence of metformin and rosiglitazone on Vitamin B12 and folic acid status and homocysteine levels was observed. In this study, under the 6-week therapy with metformin, homocysteine levels rose by 2.36 µmol/L, and blood levels of folic acid and Vitamin B12 dropped. Rosiglitazone, on the other hand, showed no impact on Vitamin B12 and folic acid status [24]. Similar results were observed in controlled interventional studies [25]. In a recent study with 126 patients with diabetes metformin treatment was associated with impaired cognitive function. Vitamin B12 and calcium supplements may alleviate metformin-induced vitamin B12 deficiency and were associated with better cognitive outcomes [26].

## 6. Groups at Risk for Vitamin B12 Deficiency

The groups at risk for a Vitamin B12 deficiency include mainly

- older persons
- vegetarians/vegans
- persons with gastrointestinal diseases
- persons with raised Vitamin B12 requirements (pregnant women, breastfeeding women, patients with autoimmune diseases or an HIV infection)
- persons under long-term treatment with proton pump inhibitors, metformin or H-2 blockers
- patients with renal diseases.

Deficiency of Vitamin B12 in elderly persons (>65 years) is due mainly to malfunction of the uptake of Vitamin B12 in the gastrointestinal tract (malabsorption). In studies of older patients with Vitamin B12 deficiency, 53% suffered from malabsorption and 33% from pernicious anaemia, and in only 2% of cases was the condition ascribed to a dietary cause. The aetiology of the Vitamin B12 deficiency remained unclear in 11% [27]. In addition, older people often have atrophic gastritis or a lack of stomach acid from other causes. Vegetarians, and vegans in particular, have an increased risk of developing a B12 deficiency in view of the fact that foods from animal sources are the main sources of Vitamin B12 [28]. In a study of lacto-vegetarians and lacto-ovo-vegetarians, 63% of the subjects showed raised methylmalonic acid levels (>271 nmol/L) and 73% reduced holotranscobalamin levels (<35 pmol/L). Vegans had raised methylmalonic acid levels in 86% of the cases and reduced holotranscobalamin levels in 90% [29]. Also particularly at risk are infants from mothers with a Vitamin B12 deficiency. These infants develop growth disorders, severe inhibition of the psychomotor development, muscular hypotonia, and brain atrophy [29]. For this reason it is essential to ensure pregnant women and nursing mothers have a sufficient intake of Vitamin B12. The importance of infestation with Helicobacter pylori or treatment with drugs such as metformin or proton pump inhibitors resulting in development of a B12 deficiency is also underestimated [2,21]. Another clinically relevant factor is “cobalamin resistance”, in which a functional Vitamin B12 deficiency can develop despite normal serum B12 values and adequate intake of Vitamin B12 with the diet [30]. This aspect must be considered in particular in older persons and patients with renal diseases and diabetes mellitus.

## 7. Prevention and Treatment of B12 Deficiency

The cause of B12 deficiency-induced haematological and neuropsychiatric diseases is the functional deficiency of Vitamin B12. This represents confirmed, internationally recognized state-of-the-art science. Therefore, diseases of this kind must be treated by administration of Vitamin B12. It has been satisfactorily demonstrated that these conditions can be effectively treated with Vitamin B12 substitution. Substitution must begin as early as possible to avoid irreversible damage. Both hydroxycobalamin and methylcobalamin and cyanocobalamin are suitable treatment. The most comprehensive catalogue of experiential data available is on cyanocobalamin.

The recommended intake of Vitamin B12 (according to the German, Austrian and Swiss Nutrition Societies, known as DACH) is 3 µg/day for adults (healthy) and 3.5–4.0 µg/day for pregnant and lactating women. In deficiencies due to alimentary problems, dosages of 10–100 µg/day lead to normalization of the levels. Higher dosages, however, are needed in cases of malabsorption, intestinal disease, or disorders affecting the utilization of Vitamin B12.

Absorption of physiological doses takes place via an active absorption mechanism. Protein-bound dietary cobalamin is released in the stomach by pepsin and hydrochloric acid and bound to an r-protein in the gastric juice produced by the salivary glands. The cobalamin is released in the duodenum and bound to intrinsic factor (IF). The IF-cobalamin complex is then absorbed in the ileum with the help of specific receptors. However, the amount absorbed through this active mechanism is limited, due to the limited intake capacity of the ileum mucosa, to 1.5 µg. This maximum amount is achieved by oral administration of 10 µg Vitamin B12. Vitamin B12 uptake is also possible by means of passive diffusion. This, however, requires higher dosages because this absorption pathway is less efficient. In cases of severe disease, especially those involving limitations on enteral intake, larger amounts of B12 are administered in initial parenteral doses to achieve a rapid onset surge [31].

### Oral or Parenteral Administration?

Parenteral administration is still the dominant method in clinical practice. The reason for this is the assumption that oral Vitamin B12 administration is ineffective. This is not in agreement with the current state of knowledge. Experimental and clinical studies have shown that treatment is also perfectly feasible with oral administration of Vitamin B12. The oral dosage required for normalization of Vitamin B12 status depends on the causes of the deficiency and the severity of the disease. In cases of deficiency due to alimentary causes, dosage 10–100 µg/day leads to normalization of the levels. However, higher dosages are needed in cases of malabsorption, intestinal disease, or disorders affecting the utilization of Vitamin B12. Randomized double-blind dosage determination studies with daily administration of 2.5, 100, 250, 500 and 1000 µg of cyanocobalamin have shown that to normalize the levels (cobalamin and holo-TC as well as MMA and homocysteine), doses of >500–1000 µg/day are required [32]. Hence, the lowest dose of oral cyanocobalamin required to normalize mild Vitamin B12 deficiency is more than 200 times the recommended dietary allowance of approximately 3 µg daily (*i*.*e*., >500 µg per day). This is also confirmed by clinical findings on factors influencing the haematological and/or neurological Vitamin B12 deficiency symptoms.

The clinical studies confirm the efficacy of orally administered cyanocobalamin in treatment of B12 deficiency-related diseases. In summing up all of the available studies, it can be assumed that in milder forms of Vitamin B12 deficiency, e.g., due to alimentary causes, doses of >100 µg/day cyanocobalamin would be recommended for prophylaxis and treatment of deficiencies and for forms resulting from absorption disorders, doses of 500–2000 µg/day would be required. According to a review by the *Cochrane Group*, the efficacy of orally administered cyanocobalamin as a curative treatment with initial doses of 1000–2000 µg daily, then weekly, is confirmed and is just as effective as parenteral administration. Initial parenteral administration is recommended only in severe neurological disorders in order to achieve a rapid onset surge of Vitamin B12 [33].

## 8. Cognitive improvement

Among the most important parameters influencing quality of life in the elderly is their nutritional and micronutrient status. For a variety of reasons, older persons are at increased risk for micronutrient deficiencies. In addition to age-related changes in organs and chronic diseases, regular intake of various medicinal drugs (such as metformin, proton pump inhibitors) also plays a role [2]. A micronutrient critical in this regard is Vitamin B12, the absorption and utilization of which is often limited in elderly subjects (>60 years). As shown by current results of an Oxford University study, improved intake of Vitamin B12 in the elderly may significantly reduce the risk of age-related brain atrophy [34].

Vitamin B12-dependent methylation reactions play a central role in intermediary metabolism. Vitamin B12 regulates, together with 5-methyl-tetrahydrofolic acid, the remethylation of homocysteine to l-methionine and the subsequent ATP-dependent formation of S-adenosylmethionine (SAM). SAM is essential to most biological methylation reactions including the methylation of myelin, neurotransmitters, and phospholipids (e.g., phosphatidylcholine). A dietary Vitamin B12 and/or folic acid deficiency is one of the most common causes of hyperhomocysteinaemia.

**Figure 3:**
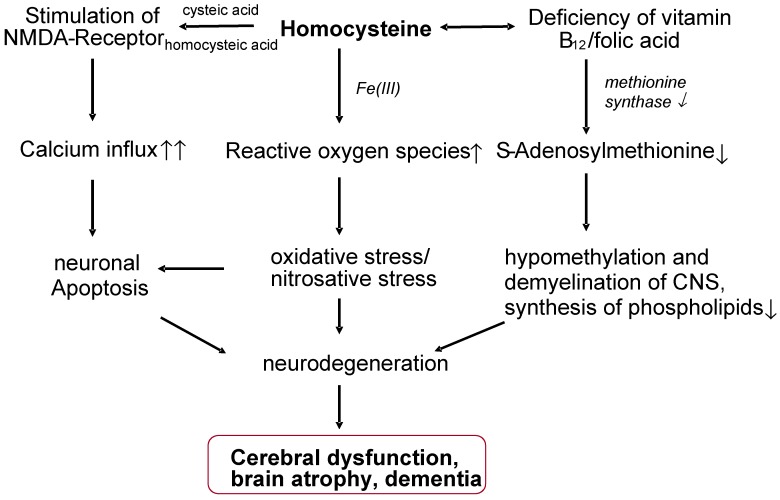
Possible neurotoxic effects of homocysteine (model).

An increase in plasma homocysteine levels is an indication of a disorder of the methyl group metabolism, which is associated with an increased risk for neuronal damage, impairment of cell proliferation, and an increased tendency to chromosome strand breaks. According to studies, a latent inadequate supply of Vitamin B12 and elevated homocysteine plasma levels (>10 µmol/L) in older persons are associated with a decline in cognitive performance [35,36,37]. The variance in cognitive performance tests in elderly subjects is attributed, regardless of the intelligence quotient, to the homocysteine levels in approximately 11% of cases [38]. The occurrence of depression in the elderly is also frequently associated with a low Vitamin B12 status and increased homocysteine levels. Hyperhomocysteinaemia, and the associated hypomethylation of the CNS, is also considered to be an independent risk factor for Alzheimer’s-type dementia (DAT) (Figure 3). In the major Framingham study, the risk of Alzheimer’s disease was nearly doubled at homocysteine levels >14 µmol/L [39]. Raised homocysteine levels are associated in elderly subjects with a smaller hippocampus and an increased rate of atrophy of the medial temporal lobe and white matter [40,41,42].

A recent prospective University of Oxford study carried out with 107 persons aged between 61 and 87 years shows a significant association between Vitamin B12 status and the size of the brain. In addition to cognitive tests and measurement of the brain with MRI, the plasma Vitamin B12 and holotranscobalamin (holoTC) levels were recorded in the healthy subjects at study onset and once a year. After 5 years, age-related brain atrophy was most advanced in the subjects with the lowest Vitamin B12 status. Compared to study participants with the highest Vitamin B12 baseline levels, the lowest tertile (plasma Vitamin B12 levels <308 pmol/L, plasma holotranscobalamin levels <54 pmol/L) was associated with a risk level raised by more than 6-fold for loss of brain volume (odds ratio 6.17; 95% confidence interval, 1.25–30.47). Of particular note is the fact that atrophy of the brain already occurred with a marginal Vitamin B12 deficiency and not at the Vitamin B12 deficiency level currently defined as manifest disease [34].

In a recent randomized and double-blind interventional study (VITACOG study) involving 168 elderly persons with mild cognitive impairment (age: >70 years), supplementation of Vitamin B12 (500 p, g/day, p.o.), folic acid (0.8 mg/day, p.o.) and vitamin B6 (20 mg/day, p.o.) over a period of 24 months significantly slowed the progression of brain atrophy and the reduction of cognitive performance (by 53.3%, *p* = 0.001) compared with the placebo group in the subjects with homocysteine levels >13 µmol/L. The accelerated rate of brain atrophy in elderly with mild cognitive impairment can be slowed by treatment with homocysteine-lowering B vitamins [43]. Vitamin B12, folic acid and vitamin B6 lower homocysteine, which directly leads to a decrease in gray matter atrophy, thereby slowing cognitive decline. B-vitamin supplementation can slow the atrophy of specific brain regions that are a key component of the Alzheimer’s disease process and that are associated with cognitive decline [44].

## 9. Conclusions

The incidence of a Vitamin B12 deficiency has to date received too little attention in orthodox medical practice. In particular, older persons, patients on long-term medication and those with neurological disorders can all benefit from adjuvant Vitamin B12 administration. Cognitive performance can be improved, and the risk of brain atrophy reduced, by Vitamin B12.

## References

[1] Gröber U. (2009). Micronutrients: Metabolic Tuning—Prevention—Therapy. Drug Metab. Drug Interact..

[2] Gröber U. (2006). Interactions between drugs and micronutrients. Med. Monatsschr. Pharm..

[3] Allen L.H. (2009). How common is vitamin B-12 deficiency?. Am. J. Clin. Nutr..

[4] (1998). Standing Committee on the Scientific Evaluation of Dietary Reference Intakes and Its Panel on Folate, Other B Vitamins, and Choline and Subcommittee on Upper Reference Levels of Nutrients; Food and Nutrition Board; Institute of Medicine. Dietary Reference Intakes for Thiamin, Riboflavin, Niacin, Vitamin B6, Folate, Vitamin B12, Pantothenic Acid, Biotin, and Choline.

[5] Healton E.B., Savage D.G. (1991). Neurologic aspects of cobalamin deficiency. Medicine (Baltimore).

[6] Neurologic Manifestations Related to Deficiency of Vitamin B12. http://yassermetwally.com/blog/?p=194.

[7] Sethi N.K., Robilotti E., Sadan Y. (2005). Neurological manifestations of Vitamin B12 deficiency. Internet J. Nutr. Wellness.

[8] Lorenzl S., Vogeser M., Müller-Schunk S. (2003). Clinically and MRI documented funicular myelosis in a patient with metabolical Vitamin B12 deficiency but normal Vitamin B12 serum level. J. Neurol..

[9] Dror D.K., Allen L.H. (2008). Effect of Vitamin B12 deficiency on neurodevelopment in infants: Current knowledge and possible mechanisms. Nutr. Rev..

[10] Dali-Youcef N., Andres E. (2009). An update on cobalamin deficiency in adults. QJM.

[11] Herrmann W., Obeid R. (2003). Functional Vitamin B12 deficiency and determination of holotranscobalamin in populations at risk. Clin. Chem. Lab. Med..

[12] Nexo E., Hoffmann-Lucke E. (2011). Holotranscobalamin, a marker of vitamin B-12 status: Analytical aspects and clinical utility. Am. J. Clin. Nutr..

[13] Schrempf W., Eulitz M., Neumeister V., Siegert G., Koch R., Reichmann H., Storch A. (2011). Utility of measuring Vitamin B12 and its active fraction, holotranscobalamin, in neurological Vitamin B12 deficiency syndromes. J. Neurol..

[14] Lindenbaum J., Savage D.G. (1990). Diagnosis of cobalamin deficiency: II. Relative sensitivities of serum cobalamin, methylmalonic acid, and total homocysteine concentrations. Am. J. Hematol..

[15] Troxler H., Hersberger M., Baumgartner M. (2008). Methylmalonsäure zur bestimmung des Vitamin B12-mangels. Schweiz. Med. Forum.

[16] Savage D.G., Lindenbaum J. (1994). Sensitivity of serum methylmalonic acid and total homocysteine determinations for diagnosing cobalamin and folate deficiencies. Am. J. Med..

[17] Nationale Verzehrsstudie II (National Nutrtion Survey II), 2008. http://www.nationale-verzehrsstudie.de.

[18] Herrmann W., Obeid R. (2005). The usefulness of holotranscobalamin in predicting Vitamin B12 status in different clinical settings. Curr. Drug Metab..

[19] Coelhoo D., Suormala T., Stucki M., Lerner-Ellis J.P., Rosenblatt D.S., Newbold R.F., Baumgartner M.R., Fowler B. (2008). Gene identification for the cblD defect of Vitamin B12 metabolism. N. Engl. J. Med..

[20] Obeid R., Schorr H., Eckert R., Herrmann W. (2004). Vitamin B12 status in the elderly as judged by available biochemical markers. Clin. Chem..

[21] Kaptan K., Beyan C., Ural A.U. (2000). Helicobacter pylori—Is it a novel causative agent in Vitamin B12 deficiency?. Arch. Intern. Med..

[22] Seal E.C., Metz J., Flicker L. (2002). A randomized, double-blind, placebo-controlled study of oralVitamin B12 supplementation in older patients with subnormal or borderline serum Vitamin B12 concentrations. J. Am. Geriatr. Soc..

[23] De Jager J., Kooy A., Lehert P. (2010). Long term treatment with metformin in patients with type 2 diabetes and risk of vitamin B-12 deficiency: Randomised placebo controlled trial. BMJ.

[24] Sahin M. (2007). Effects of metformin or rosiglitazone on serum concentrations of homocysteine, folate, and Vitamin B12 in patients with type 2 diabetes mellitus. J. Diabetes Complicat..

[25] Ting R.Z. (2006). Risk factors of vitamin B(12) deficiency in patients receiving metformin. Arch. Intern. Med..

[26] Moore E.M., Mander A.G., Ames D., Kotowicz M.A., Carne R.P., Brodaty H., Woodward M., Boundy K., Ellis K.A., Bush A.I. (2013). Increased risk of cognitive impairment in patients with diabetes is associated with metformin. Diabetes Care.

[27] Henoun Loukili N., Noel E. (2005). Cobalamin deficiency due to non-immune atrophic gastritis in elderly patients. A report of 25 cases. J. Nutr. Health Aging.

[28] Herrmann W., Schorr H. (2003). Vitamin B-12 status, particularly holotranscobalamin II and methylmalonic acid concentrations, and hyperhomocysteinemia in vegetarians. Am. J. Clin. Nutr..

[29] Lücke T., Korenke G.C. (2007). Mütterlicher Vitamin-B_12_-Mangel: Ursache neurologischer Symptomatik im Säuglingsalter. Z. Geburtshilfe Neonatol..

[30] Solomon L.R. (2007). Disorders of cobalamin (Vitamin B12) metabolism: Emerging concepts in pathophysiology, diagnosis and treatment. Blood Rev..

[31] Pietrzik K., Golly I., Loew D. (2008). Handbuch der Vitamine. Für Prophylaxe, Beratung und Therapie.

[32] Eussen S.J., de Groot L.C. (2005). Oral cyanocobalamin supplementation in older people with Vitamin B12 deficiency: A dose-finding trial. Arch. Intern. Med..

[33] Vidal-Alaball J., Butler P.P. (2009). Oral Vitamin B12 *versus* intramuscular Vitamin B12 for Vitamin B12 deficiency. Cochrane Database Syst. Rev..

[34] Vogiatzoglou A. (2008). Vitamin B12 status and rate of brain volume loss in community-dwelling elderly. Neurology.

[35] Riggs K.M. (1996). Relations of Vitamin B-12, Vitamin B-6, folate, and homocysteine to cognitive performance in the Normative Aging Study. Am. J. Clin. Nutr..

[36] Blasko I., Hinterberger M., Kemmler G. (2012). Conversion from mild cognitive impairment to dementia: Influence of folic acid and Vitamin B12 use in the VITA cohort. J. Nutr. Health Aging.

[37] Vogiatzoglou A., Smith A.D., Nurk E. (2013). Cognitive function in an elderly population: Interaction between Vitamin B12 status, depression, and apolipoprotein E s4: The Hordaland Homocysteine Study. Psychosom. Med..

[38] Budge M. (2000). Plasma total homocysteine and cognitive performance in a volunteer elderly population. Ann. N. Y. Acad. Sci..

[39] Seshadri S. (2002). Plasma homocysteine as a risk factor for dementia and Alzheimer’s disease. N. Engl. J. Med..

[40] Den Heijer H. (2003). Homocysteine and brain atrophy on MRI of non-demented elderly. Brain.

[41] Williams J.H. (2002). Minimal hippocampal width relates to plasma homocysteine in community-dwelling older people. Age Ageing.

[42] Scott T.M. (2004). Homocysteine and B vitamins relate to brain volume and white-matter changes in geriatric patients with psychiatric disorders. Am. J. Geriatr. Psychiatry.

[43] Smith A.D. (2010). Homocysteine-lowering by B vitamins slows the rate of accelerated brain atrophy in mild cognitive impairment: A randomized controlled trial. PLoS One.

[44] Douaud G., Refsum H., de Jager C.A., Jacoby R., Nichols T.E., Smith S.M., Smith A.D. (2013). Preventing Alzheimer’s disease-related gray matter atrophy by B-vitamin treatment. Proc. Natl. Acad. Sci. USA.

